# Equipping *Saccharomyces cerevisiae* with an Additional Redox Cofactor Allows F_420_-Dependent Bioconversions in Yeast

**DOI:** 10.1021/acssynbio.3c00718

**Published:** 2024-02-12

**Authors:** Misun Lee, Marco W. Fraaije

**Affiliations:** Molecular Enzymology Group, University of Groningen, Nijenborgh 4, 9747AG Groningen, The Netherlands

**Keywords:** F_420_, F_420_ biosynthesis, S. cerevisiae, tetracycline biosynthesis, F_420_-dependent bioconversion

## Abstract

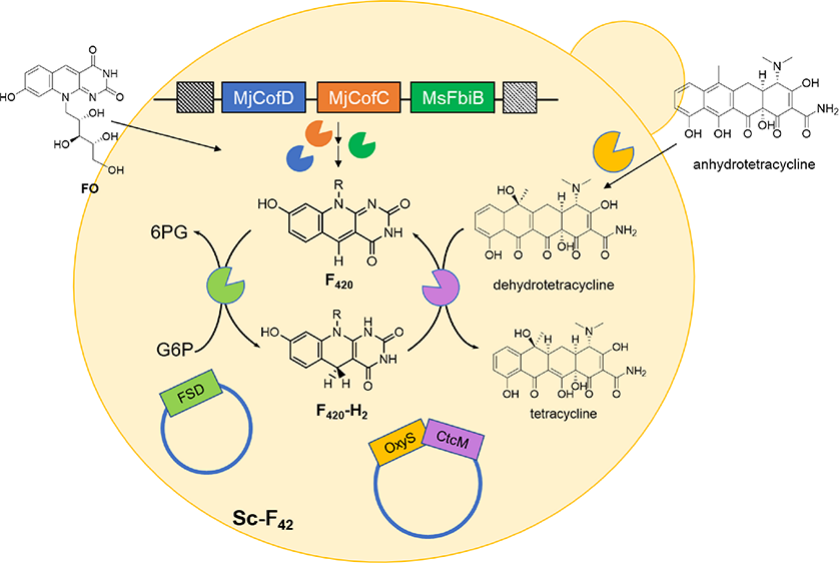

Industrial application of the natural deazaflavin cofactor F_420_ has high potential for the enzymatic synthesis of high value compounds. It can offer an additional range of chemistry to the use of well-explored redox cofactors such as FAD and their respective enzymes. Its limited access through organisms that are rather difficult to grow has urged research on the heterologous production of F_420_ using more industrially relevant microorganisms such as *Escherichia coli*. In this study, we demonstrate the possibility of producing this cofactor in a robust and widely used industrial organism, *Saccharomyces cerevisiae*, by the heterologous expression of the F_420_ pathway. Through careful selection of involved enzymes and some optimization, we achieved an F_420_ yield of ∼1.3 μmol/L, which is comparable to the yield of natural F_420_ producers. Furthermore, we showed the potential use of F_420_-producing *S. cerevisiae* for F_420_-dependent bioconversions by carrying out the whole-cell conversion of tetracycline. As the first demonstration of F_420_ synthesis and use for bioconversion in a eukaryotic organism, this study contributes to the development of versatile bioconversion platforms.

## Introduction

F_420_ is a naturally occurring deazaflavin cofactor synthesized only by certain bacteria and archaea, such as actinobacteria and methanogenic archaea.^1^ While having a similar structure as the ubiquitous flavin cofactor FAD, the chemical properties of F_420_ are more like nicotinamide cofactors, as it exclusively performs hydride transfer reactions due to the C5 of the 5-deazaiso-alloxazine moiety. F_420_-dependent reductases catalyze the asymmetric reductions of imines, ketones, enoates, etc. and can potentially be used as an alternative to flavin-containing and other NAD(P)H-dependent reductases.^2−4^ Furthermore, the low redox potential of F_420_ compared to the flavin cofactors FMN and FAD, and even to NAD(P)H, allows the reduction of recalcitrant substrates, expanding the scope of the currently available applications of enzymatic reductions.^2,5^

Despite the potential use of F_420_ for various industrial applications, the biosynthesis of this cofactor is limited to the use of natural producers such as *Mycobacterium smegmatis*, which hinders the cofactor availability and thus the related research. Therefore, its heterologous production in more versatile organisms such as *Escherichia coli* and yeast can be an attractive solution for easy access to this deazaflavin cofactor. Previous studies have shown that it is possible to produce F_420_ and analogues in *E. coli* by heterologous expression of the F_420_ biosynthetic pathway, demonstrating the potential use of this organism for the cofactor production as well as F_420_-dependent bioconversion.^6−9^ As a substitute to F_420_, a synthesis of structurally much simpler and yet functional non-natural deazaflavin analogue FOP has also been explored using both *E. coli* and *Saccharomyces cerevisiae*, offering an attractive alternative solution.^10^

Either naturally or non-naturally, F_420_ has so far been synthesized only in prokaryotic organisms. In this study, we explored the biosynthesis of F_420_ in *S. cerevisiae* to extend the F_420_-dependent biosynthesis platform even further to eukaryotic organisms. *S. cerevisiae* is a widely used organism across laboratories and industries due to its robust and harmless nature as well as its well-understood biophysical properties and well-developed molecular biological tools. Therefore, producing F_420_ in this versatile organism can expand biotechnological means for related research and applications.

**Figure 1:**
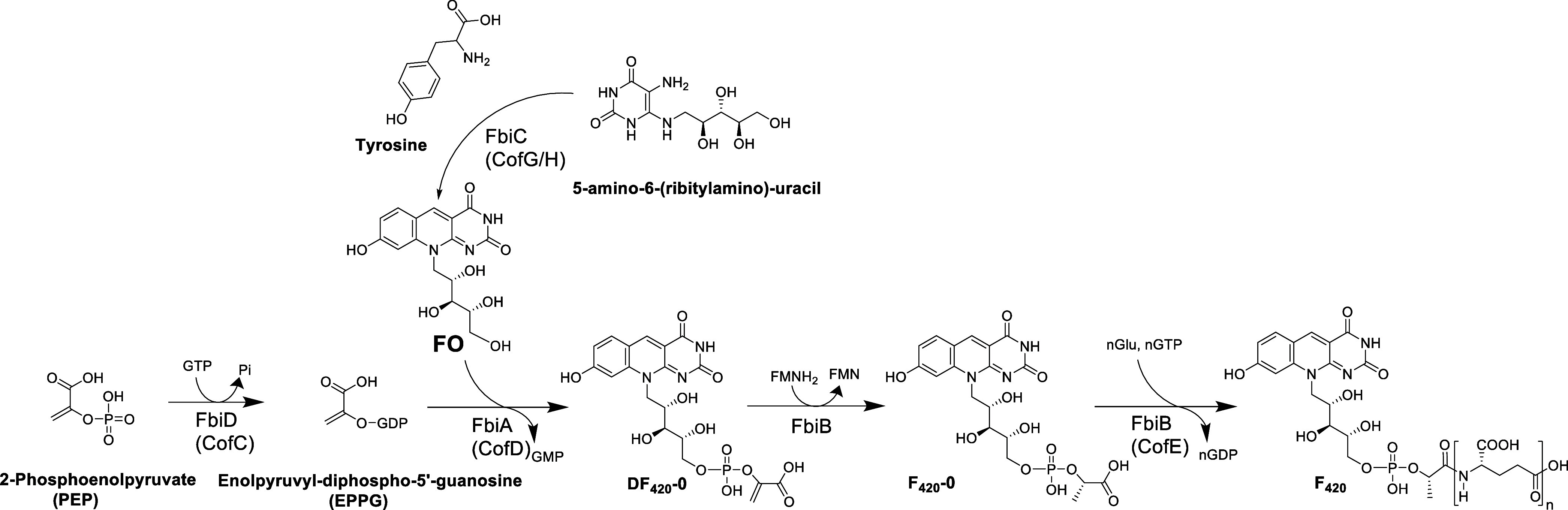
Biosynthetic pathway of F_420_. The scheme is adapted from Bashiri et al.^6^ The pathway represents the F_420_ synthesis using 2-phosphenolpyruvate (PEP) as a precursor. F_420_ can also be synthesized using 2-phospho-l-lactate (2-PL) or 3-phospho-d-glycerate (3-PG), in which case the dehydro-F_420_-0 is not formed, therefore no reduction step to F_420_-0 is required.^6,7^ Generally, the Fbi-prefix is used for the enzymes from mycobacteria, and Cof-represents the homologues from archaea.

The biosynthetic pathway of F_420_ (Figure 1) is now well-elucidated and information on the chemical and structural properties of the involved enzymes from a few representative organisms such as *M. smegmatis* and *Methanocaldococcus jannaschii* are available.^6,7,11−14^ Using the available information, we explored the use of these enzymes for the production of F_420_ in *S. cerevisiae* by testing the expression and their in vivo functions. Initial studies were performed using plasmid-based expression of the involved enzymes, and we confirmed that expression of CofC (guanylyltransferase) and CofD (FO transferase) from *M. jannaschii* along with FbiB (glutamyl ligase) from *M. smegmatis* can produce F_420_ in a good yield when the precursor FO is provided. Based on this result, we constructed a F_420_-producing *S. cerevisiae* strain by CRISPR-Cas mediated genomic integration of the three genes. The F_420_ yield after some optimization was comparable to the yield from natural producer *M. smegmatis*.

We then further explored the F_420_-producing *S. cerevisiae* strain for use in F_420_-dependent bioconversions. To demonstrate that the attained intracellular levels of F_420_ in *S. cerevisiae* can support new metabolic activities, we introduced several bacterial enzymes that catalyze the last steps of tetracycline synthesis. These involve the selective reduction of the C5a–C11a double bond of dehydrotetracycline, which requires reduced F_420_ as electron donor.^15^ By expressing the last two enzymes of the tetracycline biosynthesis as well as an F_420_-reducing enzyme from *Cryptosporangium arvum* (FSDcryar, an F_420_-dependent sugar-6-phosphate dehydrogenase)^16^ in the F_420_-producing *S. cerevisiae* strain, we could successfully convert anhydrotetracycline into tetracycline. This clearly shows the potential use of the strain for F_420_-dependent bioconversions.

Overall, the significance of the current study lies in the demonstration of the first eukaryotic production of F_420_ and the F_420_-dependent bioconversions using yeast. In addition to the previously reported *E. coli*-based production of the cofactor, this will expand the tools for F_420_-related research.

## Results and Discussion

### In Vivo FO Synthesis in *S. cerevisiae* is Hindered by Deficient Expression of FO Synthases

The catalytic core of F_420_, the 7,8-didemethyl-8-hydroxy-5-deazariboflavin (FO) moiety, is synthesized from tyrosine and 5-amino-6-(ribitylamino)-uracil. The reaction is performed by an FO synthase which mostly is (1) a bifunctional enzyme (FbiC) in actinobacteria, or (2) involves two separate enzymes (CofG and CofH) in Archaea.^1^ For in vivo production of FO, we attempted the expression of several FO synthases from different organisms including FbiCs from *M. smegmatis* (MsFbiC), *M. tuberculosis* (MtFbiC), and a eukaryote *Chlamydomonas reinhardtii* (CrFbiC)—some eukaryotes use FO as a chromophore in the DNA repair process^17^ —as well as CofG and CofH from *M. jannaschii* (mjCofG and mjCofH). The codon-optimized FO synthases were transformed in *S. cerevisiae*, and in vivo FO synthesis was analyzed. However, none of the FO synthases seem to express or function in *S. cerevisiae*, as no FO was detected in the cell extracts or in the culture media after the growth of the cells. Supplementing the growth media with tyrosine and methionine which are the precursors of FO and the cofactor SAM, respectively, did not change the result. SDS-PAGE analysis revealed no apparent protein bands for expressed FO synthases except for MjCofG (Figure S1). Previous studies showed that in *E. coli* very low expression of FO synthases was sufficient for the in vivo FO production.^6,10^ It is plausible that the expression of the SAM-dependent Fe–S cluster-containing enzymes can be problematic due to the different Fe–S cluster assembly pathways in prokaryotes and eukaryotes, causing the disturbed expression or malfunction of the enzyme.^18,19^ As the functional expression of the different FO synthases failed, we have focused on building the F_420_ pathway using the chemically synthesized FO. This would be analogous to the use of riboflavin as a precursor for flavin cofactors.

### Coexpression of CofC and CofD Enables the In Vivo Production of Dehydro F_420_ (DF_420_) in *S. cerevisiae*

The precursor in F_420_ biosynthesis was initially identified to be 2-PL but in recent studies, PEP was also shown to be a precursor in some organisms.^6,7^ When PEP is used as a precursor, dehydroF_420_-0 (DF_420_-0) instead of F_420_-0 is produced and subsequently reduced to F_420_-0 by an FMN-dependent catalysis (Grinter 2020).^11^ In some organisms, yet another precursor, 3-PG, was found to be used as the precursor, which results in the production of a F_420_ analogue, 3-PGF_420_.^7^ The substrate specificities of the enzymes involved in attaching these moieties to FO, i.e., guanylyltransferase (CofC or FbiD) and FO transferase (CofD or FbiA), are therefore different between enzyme homologues.^12^ Since 2-PL is not known to be a common metabolite in *S. cerevisiae*, a guanylyltransferase and a FO transferase that are active on the more accessible substrates PEP and EGGP, respectively, seem more suited for in vivo F_420_ synthesis in *S. cerevisiae*.

**Figure 2:**
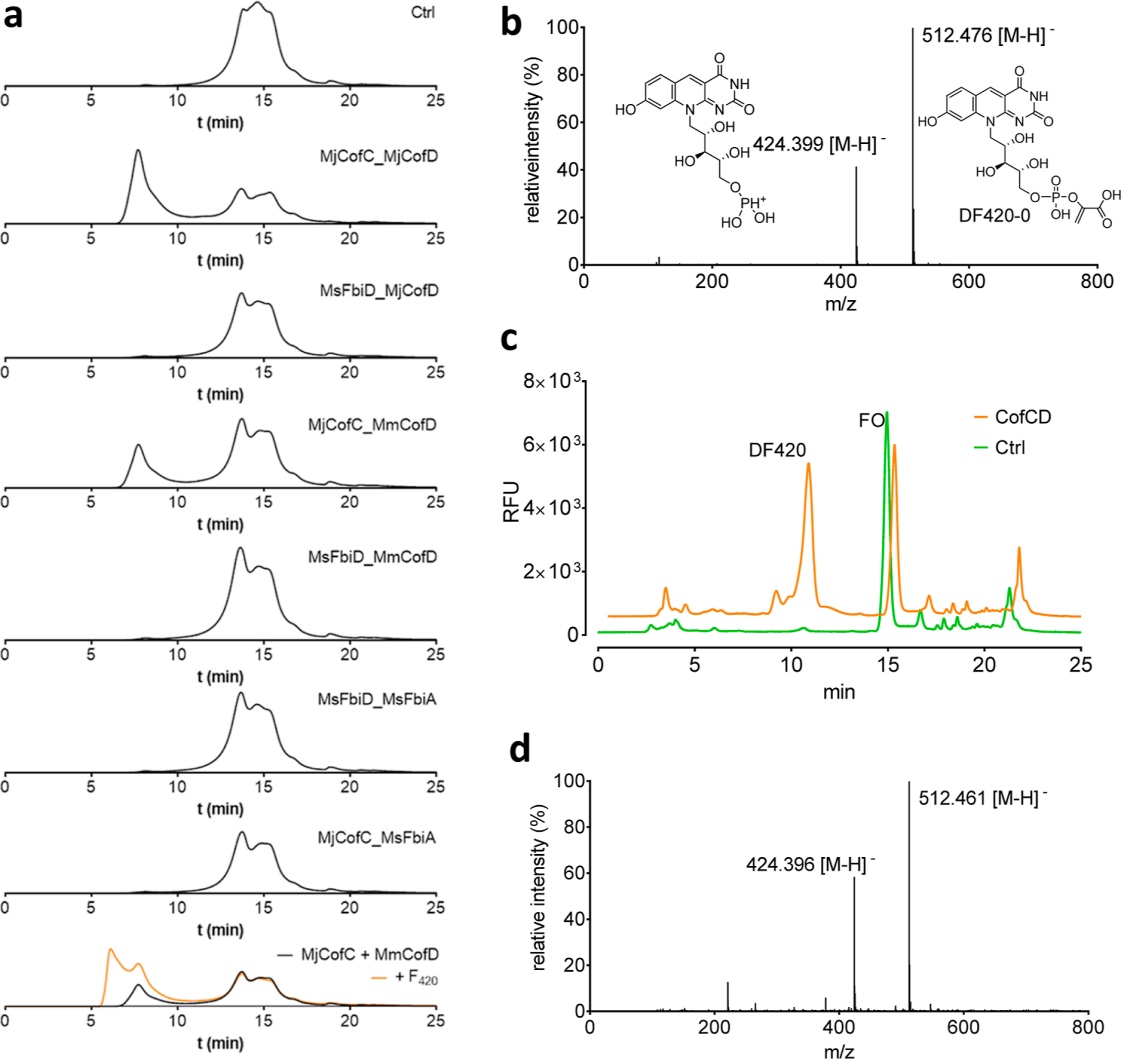
DehydroF_420_(DF_420_) production in *S. cerevisiae*. (a) HPLC analysis of in vitro reaction using *S. cerevisiae* cell extracts expressing various CofC (FbiD) and CofD (FbiA). The control reaction was performed with wild-type *S. cerevisiae* cells containing an empty plasmid. The orange chromatogram shows the reaction product of MjCofC and MmCofD, which was spiked with purified F_420_. (b) LC–MS analysis shows DF_420_-0 as the reaction product of MjCofC–MjCofD and its fragmented ion with *m*/*z* of 424.399 [M – H]^−^. (c) The HPLC result and d. LC–MS identification of in vivo DF_420_-0 production was performed using *S. cerevisiae* expressing MjCofC and MjCofD. The calculated mass of DF_420_-0 is 513.350. The ionized molecule with a *m*/*z* of 424.396 [M – H]^−^ is expected to be a fragment of DF_420_-0 as shown in panel b.

Based on the available data from previous studies, we selected two guanylyltransferases from *M. smegmatis* and *M. jannaschii* (MsFbiD and MjCofC, respectively) as well as three FO transferases from *M. smegmatis*, *M. jannaschii,* and *M. mazei* (MsFbiA, MjCofD, and MmCofD, respectively). In order to select the best combination of two enzymes for the in vivo synthesis of DF_420_-0, we first expressed these enzymes individually in *S. cerevisiae* and performed reactions with mixtures of cell extracts. Among the possible six combinations of the cell extract mixtures of guanylyltransferase and FO transferase, the combination of MjCofC–MjCofD and MjCofC–MmCofD seemed to function, showing an additional peak when compared with the control reaction on HPLC analysis (Figure 2a). The F_420_ spiked reaction product of MjCofC–MmCofD shows that the additional peak is from a potentially F_420_-like product. The products were purified and further analyzed using LC–MS, which indeed showed the mass corresponding to that of DF_420_-0 (Figure 2b). Previously it was shown that MsFbiA exclusively uses EPPG as a substrate and MsFbiD preferably accepts PEP over 2-PL when studied in vitro.^6,12^ However, in this study, reactions with MsFbiD and/or MsFbiA did not show any products, which might be due to insufficient expression. SDS-PAGE analysis (Figure S2) showed no apparent overexpression of MsFbiA and visibly lower expression of MsFbiD compared to its homologous enzyme MjCofC. Based on this result we selected MjCofC and MjCofD for in vivo F_420_ production. When coexpressed in *S. cerevisiae*, these two enzymes produced DF_420_-0 in vivo using the FO provided in the media, which was confirmed by HPLC and LC–MS analysis (Figure 2c,d). Although it was previously suggested that mainly 2-PL was used as a precursor in archaea,^12,20^ the archaeal enzymes MjCofC and MjCofD expressed in *S. cerevisiae* seemed to exclusively use PEP, producing DF_420_-0 as the only detectable product. This result indicates that the in vivo PEP concentration in *S. cerevisiae* is sufficient for these enzymes to produce DF_420_-0 and that no or an insignificant amount of 2-PL is present in the yeast cells.

**Table 1:** Effect of Media on the F_420_ Yield^a^

	nmol/L	nmol/g DW^b^	in vivo conc (μM)^c^
SD + 2% glu	184 ± 16	95 ± 6	36 ± 2
SD + 2% glu + 80 mg/L Glu	232 ± 20	97 ± 7	37 ± 3
YPD	2590 ± 410	327 ± 52	124 ± 20
VD + 2% glu + 80 mg/L Glu	1250 ± 210	292 ± 50	110 ± 19

a The experiments were performed in biological duplicates.

b The correlation between the cell dry weight (DW) and the OD_600_ was determined (1 OD_600_unit ≈ 0.37 mg/mL) to estimate the dry weight of the culture.

c The in vivo F_420_ was estimated based on the reported cell volume per biomass of *S. cerevisiae*.^26^

### *S. cerevisiae* Strain Expressing FbiB from *M. smegmatis* along with MjCofC and MjCofD Produces F_420_-*n*

The final step of F_420_ biosynthesis is the elongation of F_420_-0 with glutamyl tails in varying length by CofE or FbiB. When PEP is used as a precursor, the reduction of the intermediate product dehydroF_420_-0 (DF_420_-0) to F_420_-0 is additionally required. In mycobacteria, the bifunctional glutamyl ligase FbiB also performs the FMN-dependent reduction of DF_420_-0 to F_420_-0^11^ in addition to the glutamyl ligation reaction. However, in organisms that use monofunctional glutamyl ligase CofE, such as methanogenic archaea, the reduction is possibly performed by a “stand-alone nitroreductase”^21^ or a yet-unknown enzyme. Considering the convenience of using the bifunctional enzyme for both the reduction of DF_420_-0 and the glutamyl ligation, we chose FbiB from *M. smegmatis* (MsFbiB) for the final step of F_420_ biosynthesis. Three enzymes, MjCofC, MjCofD, and MsFbiB, were successfully expressed in *S. cerevisiae* on two separate plasmids (MjCofC and MjCofD on one and MsFbiB on the other). Gratifyingly, when the strain was grown in the media containing FO in vivo, the production of F_420_ was detected (Figure S3a). The HPLC analysis showed several peaks of potential F_420_ species, which were confirmed by the LC–MS analysis. The mass spectroscopy data showed that the produced F_420_ species mostly contained five or six glutamyl moieties (Figure S3b).

**Figure 3:**
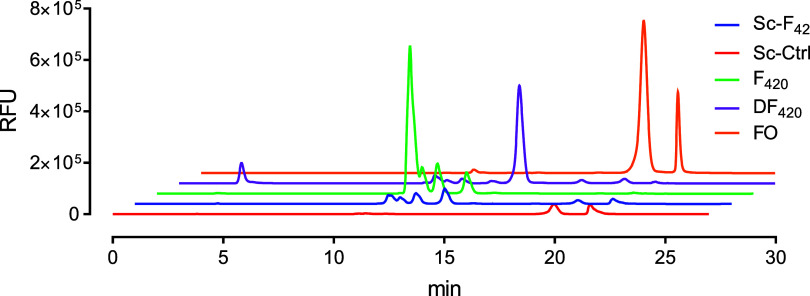
F_420_ production in *S. cerevisiae*. F_420_ purified from *M. smegmatis* is used as the standard sample. The wild-type *S. cerevisiae* grown in media containing FO is used as the control. Only the samples from strain Sc-F_420_ shows the F_420_-corresponding peaks.

After establishing a set of functional F_420_ pathway enzymes in yeast, we developed an *S. cerevisiae* strain that has a F_420_ synthetic pathway built in using CRISPR-Cas9-mediated genomic integration. One copy of each MjCofC, MjCofD, and MsFbiB genes was cloned at the HO locus as described in the Materials and Methods. The resulting strain is herein referred to as Sc-F_420_. In cell extracts of the Sc-F_420_ strain grown in synthetic defined (SD) media supplemented with FO, peaks corresponding to F_420_ were detected by HPLC-FLD, which were expectedly absent in the control sample of the wild-type *S. cerevisiae* strain (Figure 3). With the HPLC method used for verifying the in vivo F_420_ production, four apparent peaks for F_420_ were visible in both the standard samples purified from *M. smegmatis* and Sc-F_420_ samples. In order to identify the peaks, we purified the in vivo reaction products in two purification steps using anion exchange chromatography and reverse phase HPLC. The F_420_ species purified from the cell extracts using anion exchange chromatography were further separated by HPLC-FLD with an optimized elution method and collected manually. The purified F_420_ products were analyzed using LC–MS. The mass analysis confirmed that all of the peaks corresponded to F_420_ species ranging from F_420_-6 to F_420_-2 in the descending order of the retention time (Figure S4). Interestingly, the mass of one of the peaks corresponded to the unreduced (dehydro)F_420_ species with a single glutamyl tail, DF_420_-1. It shows that the glutamate ligation reaction of FbiB can occur before the reduction reaction. Confirming that all of the peaks that were detected in HPLC-FLD correspond to F_420_ species, the combined peak area was used for further quantification of F_420_ production.

In order to optimize the F_420_ yield, we tested the effect of the growth media (Table 1). 80 mg/L glutamate was added in synthetic defined media (SD and Verduyn media) to support the glutamyl ligation reaction. Among the media tested, the highest yield per culture volume was shown when a rich medium (YPD) was used. When normalized by the amount of the biomass produced, Verduyn medium (VD) with the glutamate supplement was as effective as YPD yielding around 300 nmol/g dry biomass. SD medium was least favorable for F_420_ production, yielding about 100 nmol/g dry biomass. Furthermore, when SD media was used, the strain only reached an OD_600_ of ∼5.7, while OD_600_ values of 12.8 and 23.6 were reached when VD medium or YPD medium was used, respectively. The lower cell density also contributed to the low yield per volume of culture, which improved slightly when glutamate was supplemented. The estimated in vivo concentration of F_420_ in cells grown in YPD and VD media with glutamate was similarly high, reaching over 100 μM. Therefore, while YPD would be the best choice of media for the F_420_ production purpose, VD media would be the better choice for in vivo F_420_-dependent conversion as it offers more flexible options of using selective markers for expressing additional enzymes if required. The in vivo F_420_ concentration of 100 μM would be sufficient for most F_420_-dependent reactions considering the relatively high affinity of these enzymes toward the cofactor.^22−25^

**Figure 4:**
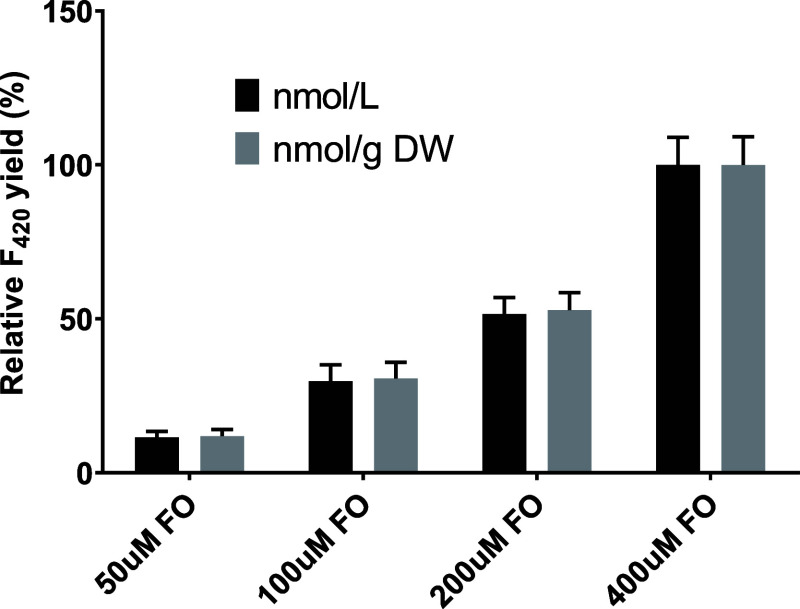
Effect of the FO concentration on the F_420_ yield.

Throughout the study, 200 μM FO was added in the media for F_420_ production. As the concentration of FO in the media can affect the physiological state of the cells as well as the F_420_ yield, we evaluated the effect of different FO concentrations on the cell growth and F_420_ production. The addition of up to 400 μM FO did not influence the growth of the Sc-F_420_ strain as the final OD_600_ upon harvest at 48 h incubation was similar, between 12 and 13, regardless of the FO concentrations added. The FO concentration showed positive correlation to the F_420_ yield as expected and this may be an indication of the low FO import efficiency into the cell (Figure 4). Even though we tested only up to 400 μM FO due to its poor solubility, it is possible that higher concentrations (if solubilized) of FO could further increase the F_420_ yield. For the purpose of F_420_ production, the highest FO concentration possible should be used, while it seems that a lower concentration (ex. 200 μM) is enough to produce sufficient in vivo F_420_ concentration for a F_420_-dependent bioconversion using cells.

The experiments are performed in duplicate, and the error bas represent the standard deviation.

In the above-described end-point measurements, a rather long incubation time of 48 h was used in order to guarantee a sufficient time for the full growth and the maximum F_420_ yield. In order to improve the efficiency of F_420_ production of the Sc-F_420_ strain, we tested the F_420_ yield at different incubation times (Table 2). Between 24 and 48 h, the cells still seem to be growing as interpreted by the increasing OD_600_. The F_420_ yield per volume culture increased between 24 and 36 h incubation time, which coincide with the increasing cell biomass. However, despite the further increased OD_600_ at 48 h, the F_420_ yield decreased slightly compared to that at 36 h incubation time. The productivity (nmol/g dry biomass), on the other hand, was the highest at 24 h showing almost 500 nmol/g DW. As a result, it also showed the highest estimated in vivo F_420_ concentration of ∼180 μM. Therefore, for further experiments, we incubated the cells only for 24 h. The F_420_ yield achieved with Sc-F_420_ is comparable to the one with *M. smegmatis* but in a much shorter incubation time (Table 3).

**Table 2:** Incubation Time-Dependent F_420_ Yield^a^

incubation time (h)	OD_600_	DW (mg)	nmol/L	nmol/g DW	in vivo conc (μM)
24	8	33	1326 ± 9	483 ± 5	183 ± 2
36	11	45	1455 ± 80	386 ± 21	146 ± 8
48	12	48	1443 ± 144	362 ± 33	137 ± 12

a The experiments were performed in triplicates. The values represent the average and the errors are the standard deviation. The yield per biomass and the in vivo F_420_ concentration were calculated as described in Table 1.

**Table 3:** F_420_ Yields Produced by Different Organisms and Conditions

	μmol/L	μmol/g DW	refs
*M. smegmatis* WT	1.43	0.3	Isabelle et al.^27^
*M. smegmatis* engineered		3	Bashiri et al.^28^
*E. coli*	0.027		Bashiri et al.^6^
*E. coli*, condition optimized	2.3	1.6	Shah et al.^8^
*E. coli*, engineered and condition optimized	11.4		Last et al.^9^
Sc-F_420_	1.3	0.48	this study

### F_420_ Producing *S. cerevisiae* Strain Sc-F_420_ Can be Used for F_420_-Dependent Bioconversion

**Figure 5:**
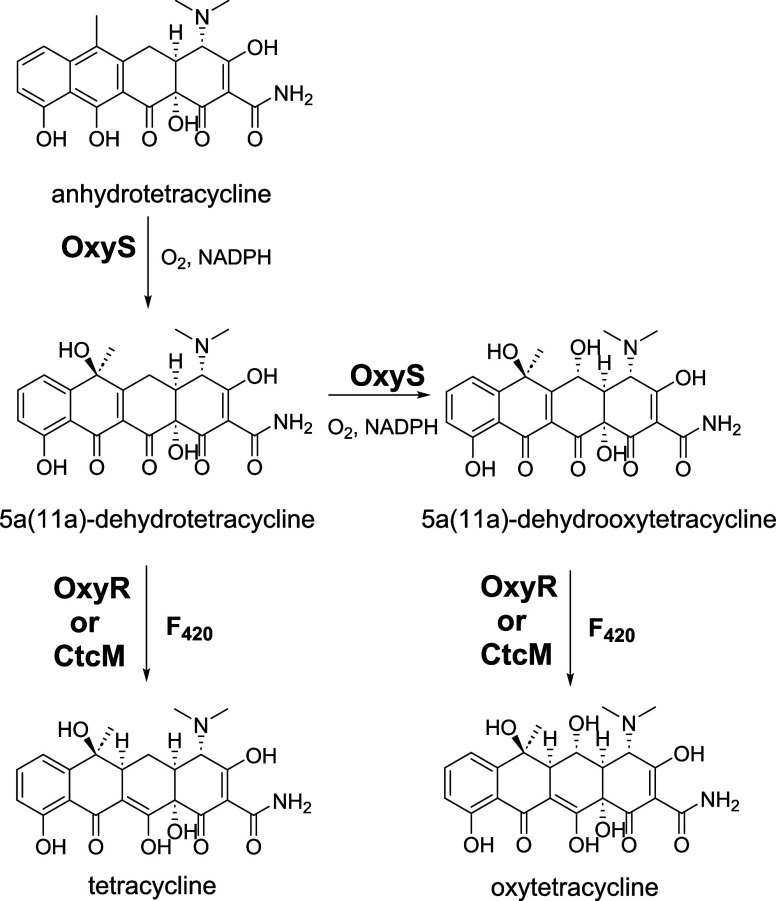
Scheme of (oxy)tetracycline synthesis from anhydrotetracycline using OxyS, OxyR, or CtcM.

Upon confirmation that in vivo F_420_ production in yeast reached high F_420_ levels, we explored the possibility to build a Sc-F_420_-based bioconversion system. The final steps of the tetracycline synthesis involve a specific F_420_-dependent enzyme-catalyzed reaction. OxyR and CtcM produce tetracycline by catalyzing the reduction at 5a(11a) of dehydrotetracycline, which is essential for the potency of the bacterial antibiotic.^29,30^ In the natural biosynthesis in *Streptomyces* species, OxyR and CtcM are known to be involved in the synthesis of oxytetracycline and chlorotetracycline, respectively.^15^ However, in vitro studies showed that both OxyR and CtcM can reduce 5a(11a)-dehydrooxytetracycline as well as 5a(11a)-dehydrotetracycline, producing oxytetracycine and tetracycline, respectively (Figure 5).^15,31^ OxyS, a flavin-dependent enzyme in the pathway, performs a single or double hydroxylation on anhydrotetracycline producing 5a(11a)-dehydrotetracycline or 5a(11a)-dehydrooxytetracycline, respectively, which can be subsequently reduced by the above-described F_420_-dependent enzymes. As a proof of concept of a Sc-F_420_-based bioconversion, we set out to demonstrate the F_420_-dependent last step of tetracycline conversion using OxyR and CtcM. As anhydrotetracycline, and not the hydroxylated product, is commercially available, we also employed OxyS for the hydroxylation reaction.

**Figure 6:**
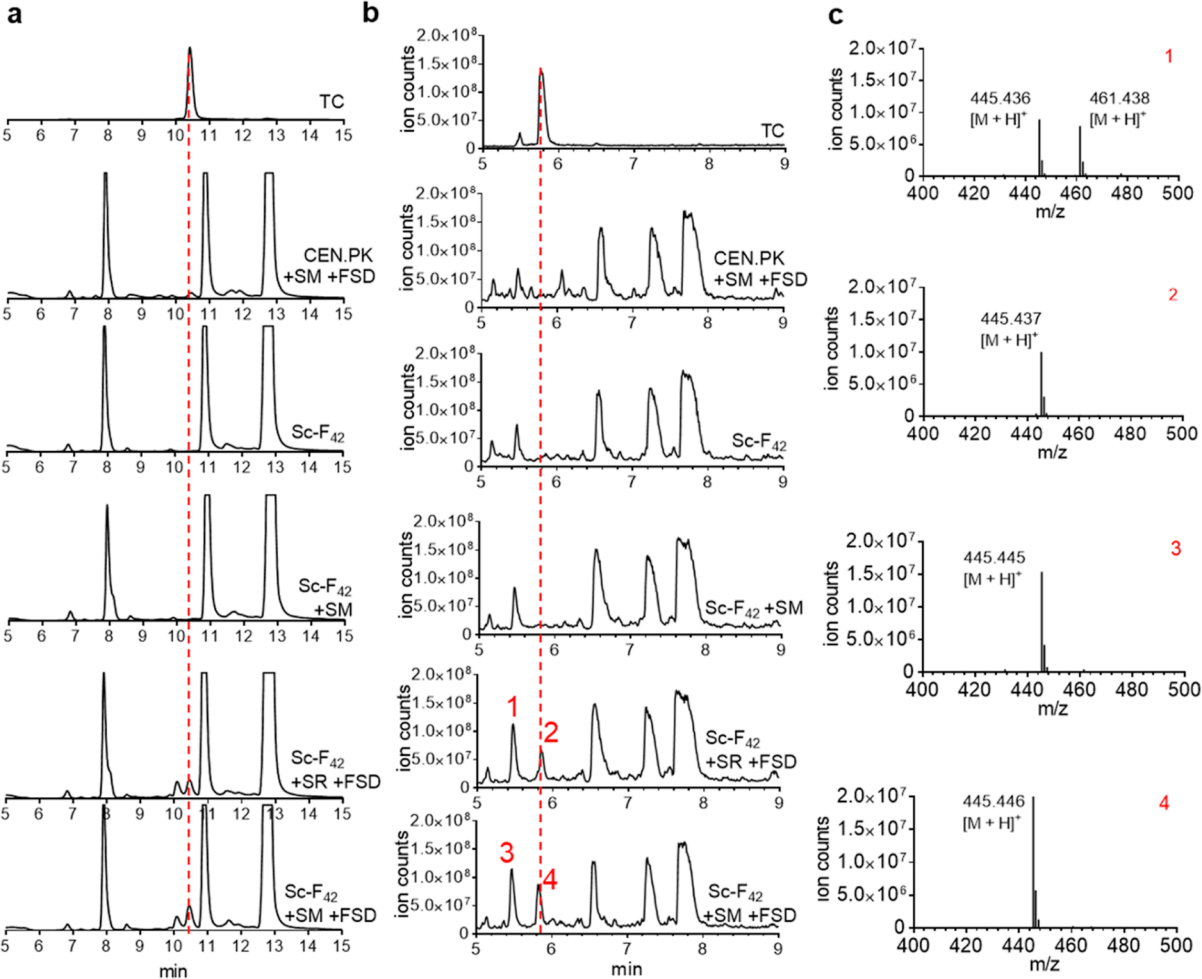
Biosynthesis of tetracycline using Sc-F_420._ HPLC (a) and LC–MS (b) analysis of production tetracycline. “SM’ and ‘SR” refer to the expression of OxyS_OxyM and OxyS_OxyR, respectively. (c) Mass spectrum of tetracycline products. The *m*/*z* of peaks 2 and 4 corresponds to tetracycline (exact mass: 444.435). Peaks 1 and 3 also show the mass that corresponds to tetracycline with slightly different *m*/*z* compared with peaks 2 and 4. This peak is also appearing in the standard tetracycline sample (b) as a minor peak. It is possible that tetracycline is epimerized in the acidic LC–MS analysis condition and eluted separately.^33^ OxyS_OxyR expressing strain also produces oxytetracycline, which is shown in peak 1, representing the [M + H]^+^ of 461.438. In contrast, OxyS_CtcM expressing strain did not produce any detectable amount of oxytetracycyline (peaks 3 and 4).

In order to produce tetracycline from anhydrotetracycline using in vivo-produced F_420_, we coexpressed OxyS together with either OxyR or CtcM on a plasmid in the Sc-F_420_ strain. For an F_420_-dependent reduction reaction, the cofactor needs to be reduced and we employed the F_420_-dependent glucose-6-phosphate dehydrogenase from *C. arvum*, expressed on a separate plasmid. This bacterial enzyme can conveniently use the available glucose-6-phosphate to generate reduced F_420_ (F_420_H_2_). After 24 h of cultivation in FO containing media and subsequent incubation with anhydrotetracycline, both OxyS_OxyR and OxyS_CtcM expressing Sc-F_420_ strains seemed to be able to produce tetracycline which was analyzed by HPLC and LC–MS methods (Figure 6). In the control reactions, wild-type CEN. PK yeast strain expressing OxyS_CtcM and FSDcryar as well as FSDcyar-absent Sc-F_420_ strain with or without OxyS_CtcM, no tetracycline related products were detected, indicating that the tetracycline was indeed produced by OxyS_OxyR or OxyS_CtcM using the in vivo produced and regenerated F_420_H_2_.

Both the Sc-F_420_ strains expressing OxyS_OxyR or OxyS_CtcM produced tetracycline but different analogues. While OxyR produced a significant portion of oxytetracycline along with the standard tetracycline, CtcM exclusively produced tetracycline and no detectable amount of oxytetracycline. This result is in line with a previous study where CtcM showed higher specificity toward dehydrotetracycline (single hydroxylation product of OxyS) thus producing tetracycline primarily.^15^ On the contrary, a previous study showed that in vitro reactions using FO and crude extract of yeast cells expressing OxyS, OxyR, and a F_420_-reducing enzyme FNO did not yield any tetracycline products.^31^ It is likely due to the nonactive OxyR when FO is used instead of F_420_. Even though it is possible to substitute F_420_ with FO or a non-natural analogue FOP for some F_420_-dependent reactions,^32^ the application is limited to the cofactor specificity of each enzyme. Therefore, conversion using the F_420_-producing system as described in this study is at an advantage for exploiting F_420_-dependent enzymes.

The production of tetracyclines in the Sc-F_420_ strain demonstrates that the strain can produce enough F_420_ for F_420_-dependent reduction and shows the potential for further exploration of the strain for F_420_-dependent bioconversions. Even though the F_420_ yield is lower than the previously constructed and engineered F_420_-producing *E. coli* strains,^8,9^ the development of the Sc-F_420_ strain as a first F_420_ producing eukaryotic organism expands the tools for F_420_ related research.

## Conclusions

F_420_ is a unique cofactor that structurally resembles the canonical flavin cofactor FAD while having a similar chemical property as the nicotinamide cofactors NAD and NADP. Despite the potential value of F_420_ for biotechnological applications, research on this cofactor and the respective F_420_-dependent enzymes has been limited by the constrained production of the cofactor using unconventional laboratory organisms such as *M. smegmatis*. In this study, we tackled this problem by producing the cofactor in a robust industrial microorganism, *S. cerevisiae*, through heterologous expression of part of the F_420_ pathway. By optimizing the enzyme combination and the growth medium, we achieved comparable F_420_ yields to that of the natural production by *M. smegmatis* but in a much shorter production time. Furthermore, we demonstrated the use of the F_420_-producing *S. cerevisiae* strain for F_420_-dependent bioconversion by showing the successful bioproduction of tetracycline from anhydrotetracycline. Together with the previously developed F_420_-producing *E. coli* strain by others, the first F_420_ producing eukaryotic strain developed in this study extends the toolbox for further development in the field of F_420_-related research.

## Materials and Methods

### Strains and Plasmids

All plasmids used in this study are *E. coli*—yeast shuttle vectors assembled using a modular cloning kit, Moclo-YTK from Addgene and the assembly was performed as described previously^34^ with some modification in the Golden Gate assembly methods. *E. coli* NEB10-beta strain was used for cloning purposes. CEN. PK2-1C strain was purchased from Euroscarf and used for constructing the F_420_-producing yeast strain.

### Growth Media

For the growth of *E. coli*, Lysogeny broth medium containing an appropriate type of antibiotic (100 μg/mL ampicillin, 50 μg/mL kanamycin, or 50 μg/mL chloramphenicol) was used. Media for *S. cerevisiae* used in this study are SD medium, YPD medium (formedium), and VD. The SD medium is composed of 6.9 g/L yeast nitrogen base without amino acids (formedium), 0.77 g/L complete supplement mixture (formedium) that is appropriate for the auxotroph markers, and 2% (w/v) glucose. VD containing 2% glucose was made according to Verduyn et al.^35^ For F_420_ production in *S. cerevisiae*, 72 mg/L FO was added to respective media prior to autoclave sterilization. FO was chemically synthesized as described in Drenth et al.^32^ For optimization of F_420_ production, 80 mg/L glutamate was added to the respective medium.

### Cloning

All *S. cerevisiae* codon-optimized genes tested for F_420_ biosynthesis and F_420_-dependent bioconversion were purchased from Twist Bioscience. The gene fragments were first cloned into an entry vector and subsequently cloned into a preassembled *E. coli*-yeast shuttle vector by Goldengate assembly method according to the Moclo-YTK cloning protocols.^34^ All cloning products were initially transformed in *E. coli* NEB10b strain using the heat shock method, isolated, and analyzed by sequencing at Eurofins. The correct cloning products were then transformed to *S. cerevisiae* CEN. PK2-1C using the lithium acetate/single-stranded carrier DNA/PEG method that is optimized by Gietz et al.^36^

### Construction of the F_420_-Producing Sc-F_420_ Strain

The genes encoding MjCofC, MjCofD, and MsFbiB were integrated in the HO locus using a Crispr-Cas9-mediated method. A vector containing an sgRNA sequence and Cas9 expression cassette was assembled using a Moclo YTK kit. The 20mer target sequence of sgRNA is 5′-GCTCCAGCATTATAGCATGC-3′. The vector contained CEN6/ARS4 origin, and pPGK1 promoter was used for Cas9 expression. The repair fragment was constructed via assembling the F_420_ pathway genes, HO locus homology fragments as well as a *leu3* into a multigene plasmid and subsequently linearizing by NotI digestion. The resulting fragment contained 5′-HO homology sequence, *cofD*, *cofC* and *fbiB*, *leu3*, as well as 3′-HO homology sequence in this order. The transformation of CEN.PK21-C strain with Cas9_sgRNA plasmid and the repair fragment was performed following the protocol of Gietz et al.^36^ Approximately 500 ng of Cas9_gRNA plasmid and 5 μg of repair fragment was used for the transformation. The PCR verification of the correct integration was performed on the genomic DNA extracted from selected colonies. Cas9-gRNA plasmid was removed from the transformants by growing the cells in nonselective YPD media and confirming the loss of uracil selectivity that the plasmid was carrying.

### In Vitro and In Vivo DF_420_ Conversion

In order to find a functional combination of PEP guanylyltransferase (FbiB/CofC) and FO transferase (FbiA/CofD) for in vivo DF_420_ production, reactions using crude extracts mixture of *S. cerevisiae* expressing each of the enzyme types were tested. Cells expressing FbiB from *M. smegmatis*, CofC from *M. jannaschii*, FbiA from *M. smegmatis*, CofD from *M. jannaschii,* or CofD from *M. mazei* were grown in 20 mL SD media with 2% glucose for 24 h at 30 °C. Cells were harvested and washed with ddH_2_0 and resuspended in 1 mL of 50 mM KPi, pH 7.0 containing 1 mg/mL Zymolyase (Amsbio). The cell resuspension was then incubated for 20 min at 30 °C and subsequently vortexed in the presence of equal volume of glass beads (five repeats of 5 s vortexing and 10 s resting on ice). Total protein concentration in the crude extracts was measured by Bradford assay. One ml reaction mixtures in 50 mM KPi, pH 7.0 containing 1 mM GTP, 1 mM phosphoenolpyruvate, 5 mM MgCl_2_, 200 μM FO and mix of CofC (FbiD)- and CofD (FbiA)-containing crude extracts (normalized to 1 mg of total protein each) were incubated at 30 °C for 4 h. The reactions were stopped by heating at 95 °C for 10 min and subsequently centrifuged and filtered. The reaction samples were analyzed by HPLC method for DF_420_ production.

### F_420_ Production Using *S. cerevisiae*

For in vivo production of F_420_ in *S. cerevisiae*, the respective cells were first grown in 5 mL of SD media with 2% glucose overnight at 30 °C. The precultures were then diluted to OD_600_ ∼ 0.2 in an appropriate FO-containing media and grown at 30 °C. Unless otherwise stated, the cells were harvested after 24 h and washed with ddH_2_O. In order to extract the in vivo produced F_420_, the cells were incubated with approximately 4× cell volume of 70% boiling ethanol at 95 °C for 5 min. The supernatants were collected after centrifugation at 8000*g* for 10 min, and the process was repeated once. The collected solutions were subjected to vacuum centrifugation at 60 °C for 1 h in order to remove ethanol and concentrate. The products were resuspended in ddH_2_O, centrifuged, and filtered prior to the analysis.

### Tetracycline Conversion

F_420_-producing yeast strain Sc-F_420_ expressing OxyR or CtcM as well as OxyS and FSDcryar were grown in 20 mL VD media containing 80 mg/L glutamate and 72 mg/L FO for 24 h in order to accumulate the in vivo F_420_. Cells were collected by centrifugation at 5000*g* for 10 min and resuspended in 1 mL of reaction solution containing 1 mM anhydrotetracycline, 5 mM glucose, and 100 mM Tris·HCl, pH 7.5. The reactions were incubated for 24 h at 30 °C and subsequently extracted three times with 2 mL of EtOAc. The reactions were dried by vacuum centrifugation for 1 h, redissolved in 1 mL of ddH_2_0, and centrifuged for 10 min at 11.000*g* to remove any debris prior to analysis.

### Analytical Methods

For all HPLC analyses, samples were separated on a Phenomenex Germini C18 (4.6 × 250 mm, 5 μm) column using a JASCO LC-4000 system equipped with an UV–vis detector and a fluorescence detector. F_420_ and intermediates were detected using UV absorbance at 262 nm and fluorescence (ex: 400 nm and em: 470 nm). For the tetracycline conversion analysis, the reactions were monitored by UV absorbance at 265 nm. The mobile phase consisting of 50 mM ammonium acetate, pH 6.0 with 5% acetonitrile (A) and 100% acetonitrile (B) was applied with a flow rate of 1 mL/min for all analysis. Slightly different elution methods were applied for each analysis, which are as follows. In vivo and in vitro DF_420_ conversion: *t* = 0 min/100:0 (A/B), *t* = 16 min/80:20 (A/B), *t* = 19 min/5:95 (A/B), *t* = 22 min/5:95 (A/B), *t* = 26 min/95:05 (A/B), and *t* = 28 min/100:0 (A/B). In vivo F_420_ production: *t* = 0 min/100:0 (A/B), *t* = 5 min/90:10 (A/B), *t* = 16 min/90:10 (A/B), *t* = 19 min/5:95 (A/B), *t* = 22 min/5:95 (A/B), *t* = 26 min/100:0 (A/B), and *t* = 28 min/100:0. For improved separation of F_420_ species: *t* = 0 min/100:0 (A/B), *t* = 5 min/92:08 (A/B), *t* = 16 min/92:08 (A/B), *t* = 19 min/5:95 (A/B), *t* = 22 min/5:95 (A/B), *t* = 26 min/100:0 (A/B), and *t* = 28 min/100:0. For the tetracycline conversion analysis, the analytes were separated using the following elution method: *t* = 0 min/100:0 (A/B), *t* = 16 min/80:20 (A/B), *t* = 19 min/05:95 (A/B), *t* = 22 min/5:95 (A/B), *t* = 26 min/100:0 (A/B), and *t* = 28 min/100:0.

All LC–MS analysis described in this study were performed on ACQUITY TQD UPLC–MS system (Waters) using the ACQUITY UPLC HSS T3 column (1.8 μm, 2.1 × 150 mm, Waters). 0.1% formic acid in water (A) and 0.1% formic acid in acetonitrile (B) were applied at a flow rate of 0.31 mL/min as the mobile phase. Following gradient methods were used for the respective analysis: DF_420_ production—*t* = 0 min/100:0 (A/B), *t* = 5 min/75:25 (A/B), *t* = 6.12 min/5:95 (A/B), *t* = 7.14 min/5:95 (A/B), *t* = 8.16 min/75:25 (A/B), and *t* = 9.18 min/100:0 (A/B); F_420_ production—*t* = 0 min/100:0 (A/B), *t* = 2 min/90:10 (A/B), *t* = 4 min/90:10 (A/B), *t* = 4–6 min/85:15 (A/B), *t* = 6–8 min/80:20 (A/B), *t* = 12 min/5:95 (A/B), *t* = 14 min/5:95 (A/B), *t* = 16 min/100:0 (A/B), *t* = 17 min/100:0 (A/B); and tetracycline conversion—*t* = 0 min/100:0 (A/B), *t* = 16 min/5:95 (A/B), *t* = 18 min/95:5 (A/B), and *t* = 20 min/100:0 (A/B). The electrospray ionization (ESI) in the negative ion mode for DF_420_ as well as F_420_ detection and positive ion mode for tetracycline detection were used.

## Supporting Information Available

SDS-PAGE analyses of enzyme expression and LC/MS analyses of the deazaflavins produced by recombinant yeast (PDF)
