# Supplementary material for: Equipping Saccharomyces cerevisiae with an Additional Redox Cofactor Allows F420-Dependent Bioconversions in Yeast
Source: ACS Synth Biol. 2024 Feb 12;13(3):921–9. doi: 10.1021/acssynbio.3c00718 (PMC10949242; doi:10.1021/acssynbio.3c00718)
Supplement: Supplementary file 1 — sb3c00718_si_001.pdf [file sb3c00718_si_001.pdf]

Supplementary information

Equipping *Saccharomyces cerevisiae* with an additional redox cofactor allows F_{420}-dependent bioconversions in yeast

Misun Lee[1‡] and Marco W. Fraaije[1*]

[1] Molecular Enzymology Group, University of Groningen, Nijenborgh 4, 9747AG Groningen, The Netherlands

‡Current address: CJ CheilJedang Corp., CJ blossom park, 1356 Iui-dong, Yeongtong-gu, Suwon, Gyeonggi-do, Korea

*Corresponding author: m.w.fraaije@rug.nl

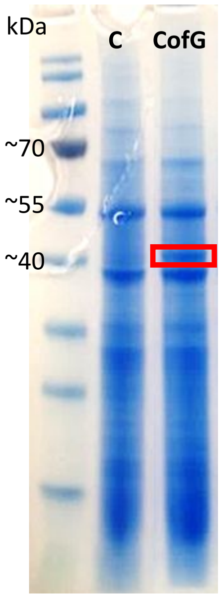

Figure S1. SDS-PAGE analysis of MjCofG expression.

The estimated size of MjCofG is approximately 41.6 kDa. The control sample is the cell extract of the wild type CEN. PK21-C strain carrying the empty vector.

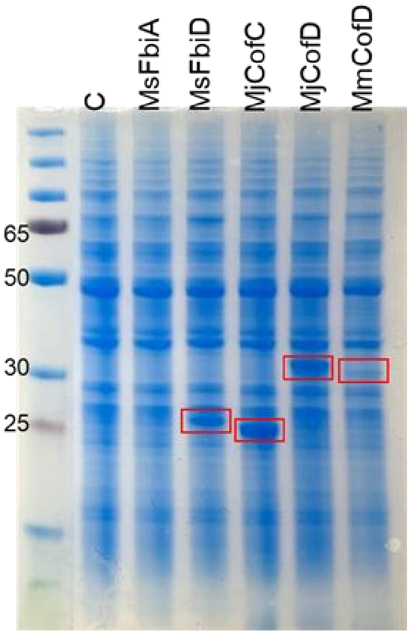

Figure S2. SDS-PAGE analysis of guanylyltransferases and FO transferases.

The estimated sizes of the enzymes are as follows: MsFbiA – 35 kDa, MsFbiD – 23 kDa, MjCofC – 26 kDa, MjCofD – 35 kDa and MmCofD – 33 kDa. The size of MsFbiD appears to be slightly bigger than the expected size which may be due to glycosylation in *S. cerevisiae*. The control sample is the cell extract of the wild type CEN. PK21-C strain carrying the empty vector.

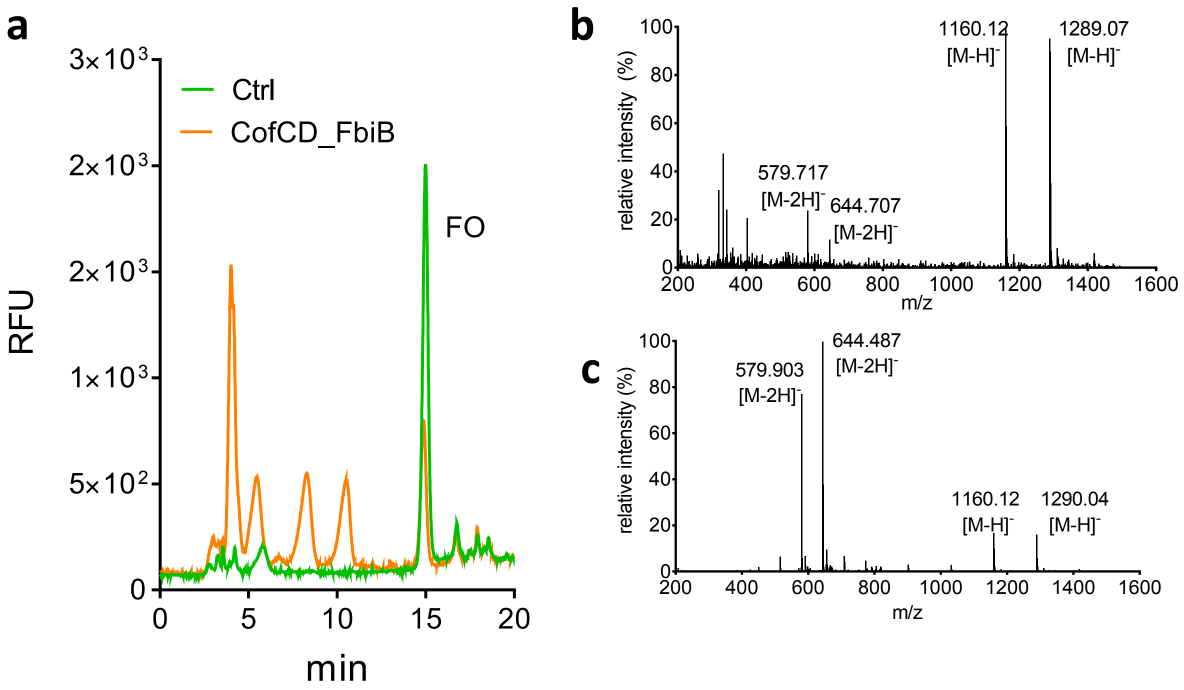

Figure S3. In vivo F_{420} synthesis of *S. cerevisiae* by plasmid-based expression of MjCofC, MjCofD and MsFbiB. a. HPLC analysis of F_{420} production: the strain expressing MjCofC, MjCofD and MsFbiB (orange line) shows a decrease of FO level and products formation compared with the control strain carrying empty vectors (green line). The LC-MS analysis of the products (b) shows the m/z of F_{420}-5 (1160.12 $[M-H]^-$) and F_{420}-6 (1289.07 $[M-H]^-$) which are comparable to that of F_{420} purified from *M. smegmatis* (c).

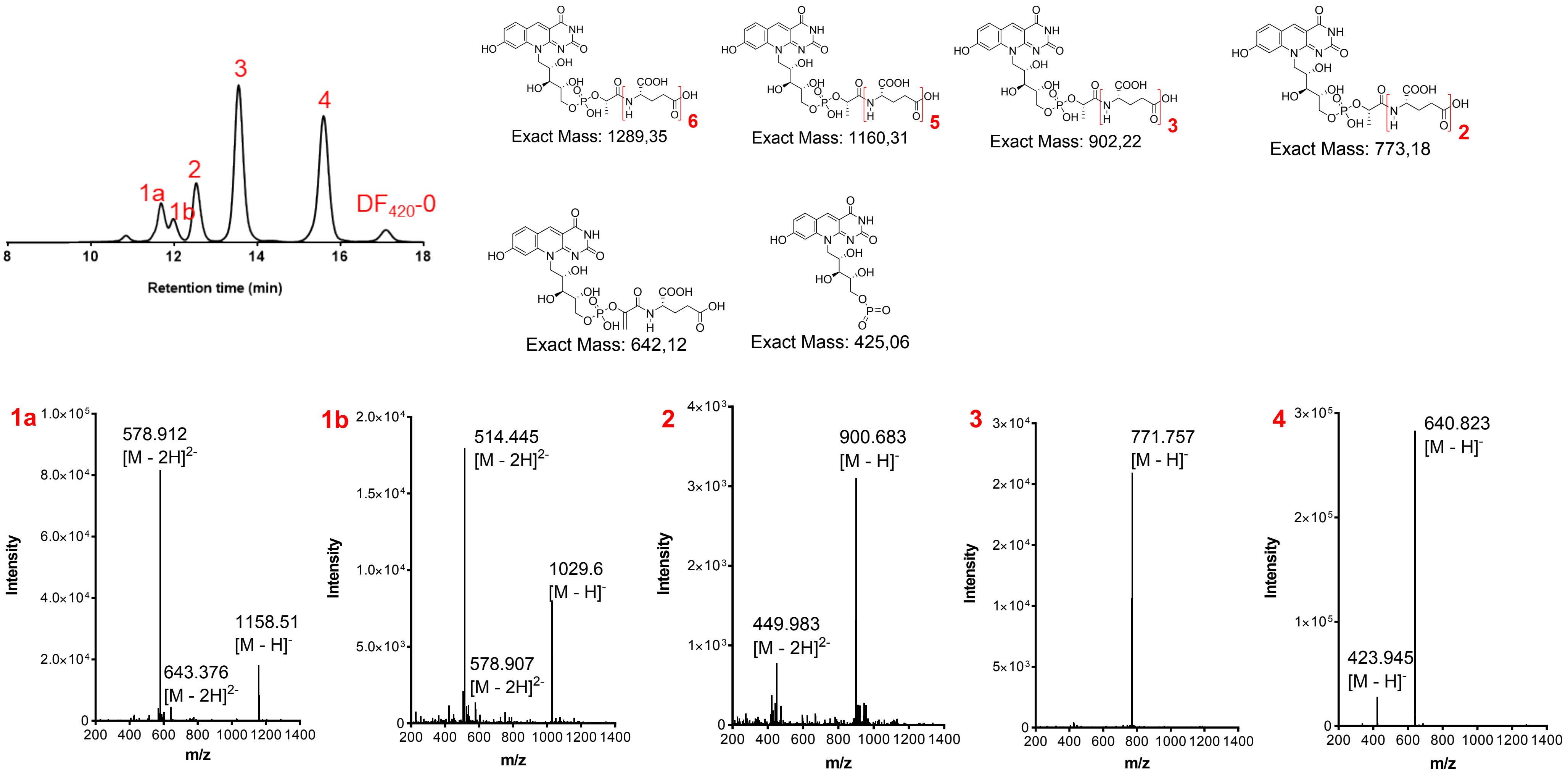

Figure S4. Analysis of F_{420} species purified from Sc-F_{420} culture grown in FO containing media.

HPLC analysis of F_{420} purified with ion-exchange chromatography shows five potential F_{420} peaks and a dehydro-F_{420}-0 (upper left chromatogram). The MS spectrometry represents the m/z of each peak which is purified and analyzed on separate runs. The m/z of peak 4 corresponds to the dehydro-F_{420}-1.
